# Supplementary material for: Gene Expression Profiling Reveals that PXR Activation Inhibits Hepatic PPARα Activity and Decreases FGF21 Secretion in Male C57Bl6/J Mice
Source: Int J Mol Sci. 2019 Aug 1;20(15):3767. doi: 10.3390/ijms20153767 (PMC6696478; doi:10.3390/ijms20153767)

SUPPLEMENTARY INFORMATION FOR

Gene expression profiling reveals that PXR activation inhibits hepatic PPARα activity and decreases FGF21 secretion in male C57Bl6/J mice

Sharon Ann Barretto [1,*], Frederic Lasserre [1,*] , Anne Fougerat [1], Lorraine Smith [1], Tiffany Fougeray [1,] , Celine Lukowicz [1], Arnaud Polizzi [1], Sarra Smati [1] , Marion Régnier [1], Claire Naylies [1], Colette Bétoulières [1], Yannick Lippi [1] , Hervé Guillou [1], Nicolas Loiseau [1], Laurence Gamet-Payrastre [1] , Laila Mselli-Lakhal [1] and Sandrine Ellero-Simatos [1,*]

Supplementary Table 1: Gene Enrichment Analysis on the 1029 hepatic genes induced by PCN-treatment only in WT mice (prototypical *Pxr* target genes, fold-change>1.5 & adjusted p-value<0.05) in the liver. See enclosed excel file.

Supplementary Table 2: Impact on PCN treatment on all XMEs in the liver and in the ileum. See enclosed excel file.

Supplementary Table 3: Primers used for qPCR

Supplementary Figure 1: Effect of PCN treatment on additional plasmatic parameters. Data are shown as mean±SEM of n=5-6 per group. *p≤0.05, **p≤0.01, ***p≤0.005 for PCN effect using 2-way ANOVA and Bonferroni's post-tests.

Supplementary Figure 2: Heatmap representing the significantly regulated genes (fold-change>1.5 & corrected p-value < 0.05). Hierarchical clustering is also shown, which allows the definition of five gene clusters. For each gene cluster, the mean expression value is shown on the box-plots.

Supplementary Figure 3: Microarray data and complementary qPCR confirmation in the liver for selected genes involved in *de novo* lipogenesis. Data are shown as mean±SEM of n=5-6 per group. *p≤0.05, **p≤0.01, ***p≤0.005 for PCN effect and #p≤0.05, ##p≤0.01, ###p≤0.005 for genotype effect using 2-way ANOVA and Bonferroni's post-tests.

Supplementary Figure 4: Microarray data and complementary qPCR confirmation in the liver for selected genes involved in (a) cholesterol synthesis and (b) fatty acid / cholesterol transport. Data are shown as mean±SEM of n=5-6 per group. *p≤0.05, **p≤0.01, ***p≤0.005 for PCN effect and #p≤0.05, ##p≤0.01, ###p≤0.005 for genotype effect using 2-way ANOVA and Bonferroni's post-tests.

Supplementary Table 3 Oligonucleotide sequences for real-time PCR

Gene	NCBI Refseq	Forward primer (5'-3')	Reverse primer (5'-3')
Abca1	NM_013454	GCGCTACAACATGGACATCCT	GCTGGGTCGGGAGATGAGA
Abcg5	NM_031884	TCGCCACGGTCATTTTCA	GCCAAAAGAGCAGCAGAGAAATA
Acly	NM_134037	AAAGCTTGGCCTCGTCGG	GGGACGAAGGGTTCAATGAGA
Acot1	NM_012006	CGATGACCTCCCCAAGAACA	CCCAAGCAGCCCAATTCC
Bmal1	NM_007489	CAAACTACAAGCCAACATTTCTATCAG	TCGGTCACATCCTACGACAAAC
Cd36	NM_007643	GTTAAACAAAGAGGTCCTTACACATACAG	CAGTGAAGGCTCAAAGATGGC
Cyp2c55	AY206875	TTGTGGAAGAGCTAAGAAAAGCAAAT	GAGCACAGCTCAGGATGAATGT
Cyp3a11	NM_007818	TCACACACACAGTTGTAGGCAGAA	GTTTACGAGTCCCATATCGGTAGAG
Cyp4a10	NM_010011	ATTAGTGAGAGTGAGGACAGCAACAG	CCAACCCGATTTGCAGACA
Cyp4a14	NM_007822	TCAGTCTATTTCTGGTGCTGTTC	GAGCTCCTTGTCCTTCAGATGGT
Cyp7a1	NM_007824	ATCAAAGAGCGCTGTCTGGGT	GCGTTAGATATCCGGCTTCAAAC
Fasn	NM_007988	AGTCAGCTATGAAGCAATTGTGGA	CACCCAGACGCCAGTGTTC
Fgf21	NM_020013	AAAGCCTCTAGGTTTCTTTGCCA	CCTCAGGATCAAAGTGAGGCG
Ldlr	NM_010700	GCAAGGACATGAGCGACGA	CTCCCCACTGTGACACTTGAAC
Lpin1	NM_172950	ATGTTTCCCATAGAGATGAGCTCG	GAATGGTGGTACATCATTAGGAAGAG
Pparα	NM_011144	CCCTGTTTGTGGCTGCTATAATTT	GGGAAGAGGAAGGTGTCATCTG
Pparγ2	NM_011146	GATGCACTGCCTATGAGCACTT	GAATGGCATCTCTGTGTCAACC
Pxr	NM_010936	AGAGATCATCCCTCTTCTGCCAC	GATCTGGTCCTCAATAGGCAGGT
Rev-erbα	NM_145434	CAGCTGGTGAAGACATGACGAC	GGAGGAGCCACTAGAGCCAA
Scd1	NM_009127	CAGTGCCGCGCATCTCTAT	CAGCGGTACTCACTGGCAGA
Sqle	NM_009270	GGAGGCTACCGTGTTCTCCA	CTGCACTTGGTTGGTTTCTGAC
Srebp1a/c	NM_011480	CAGACACTGGCCGAGATGTG	CTTGGTTGTTGATGAGCTGGAG
Srebp2	NM_033218	GTACTGCGCCCAGAGGAGC	GCCTGAGGTTTCACCAAGGAC

Supplementary Figure 1

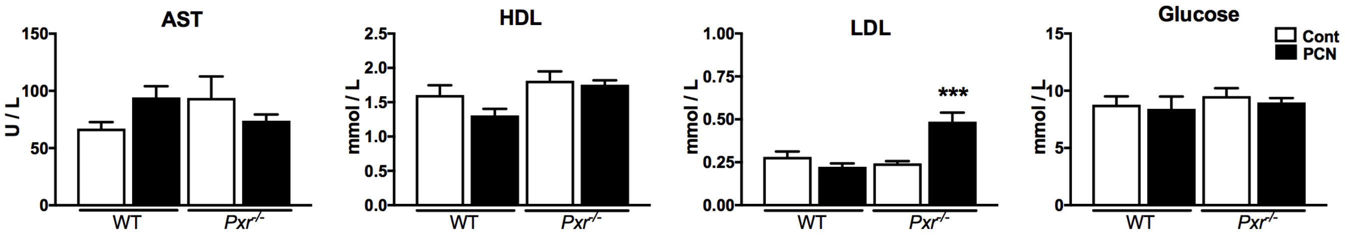

Supplementary Figure 2

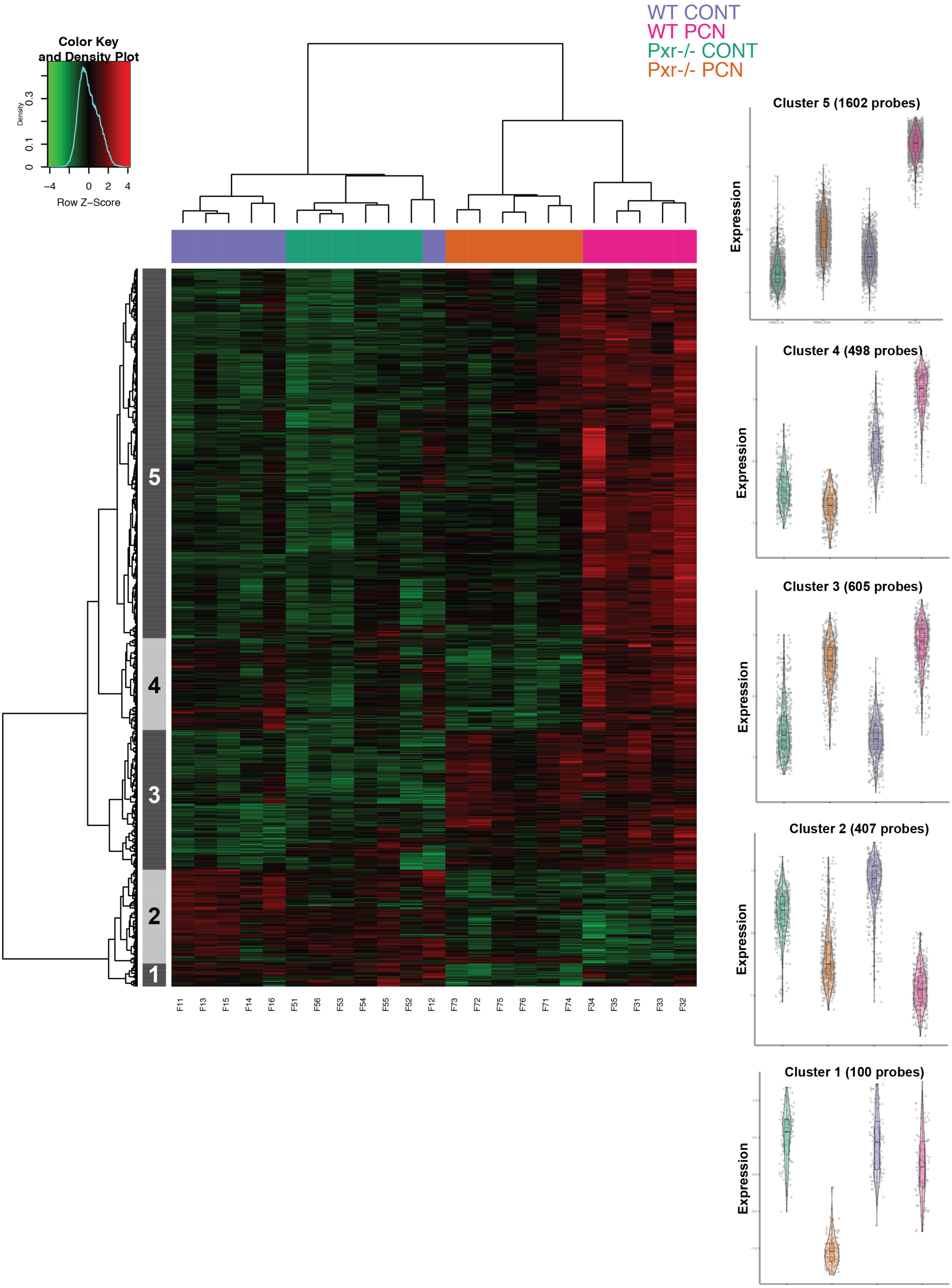

Supplementary Figure 3

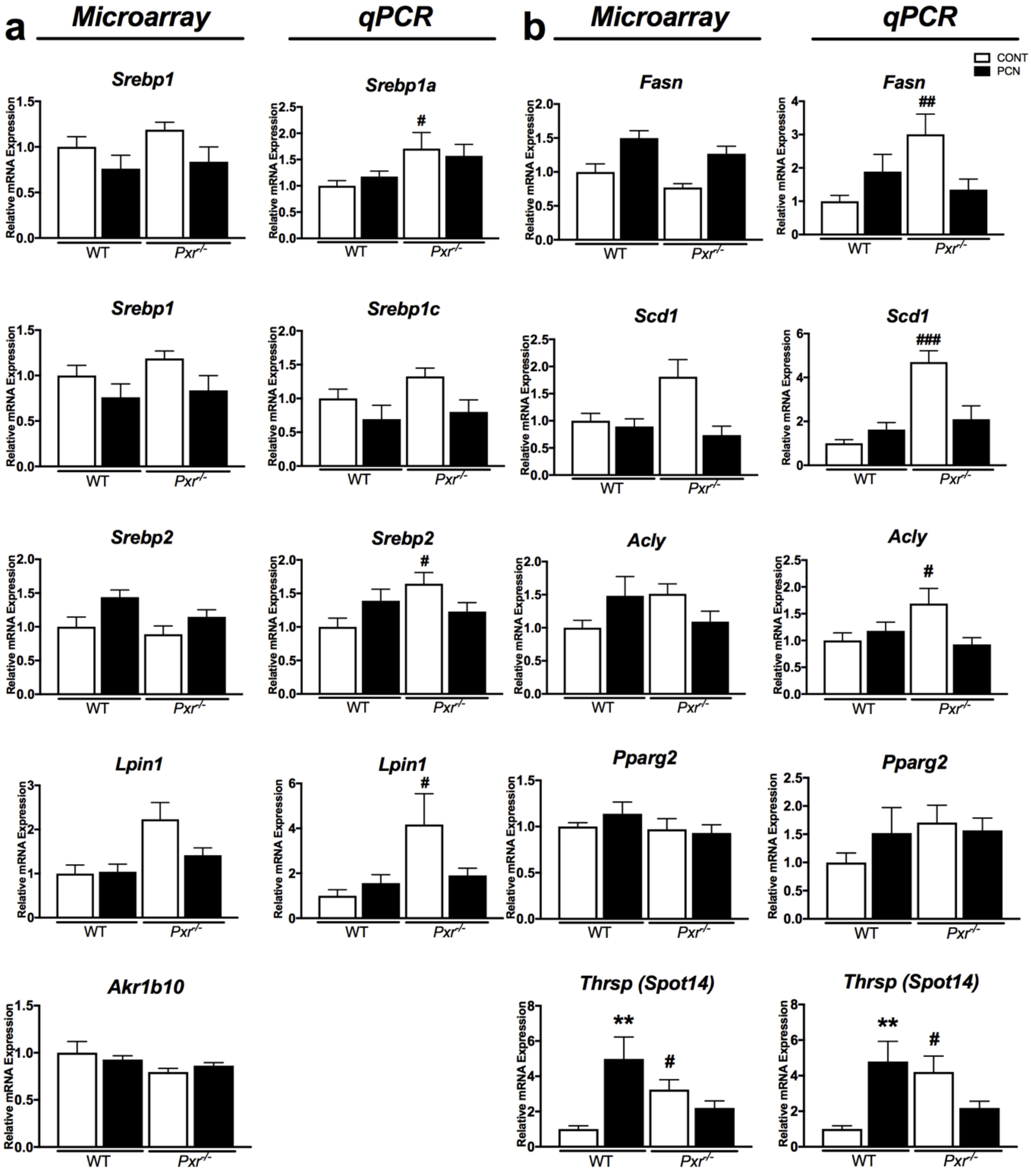

Supplementary Figure 4

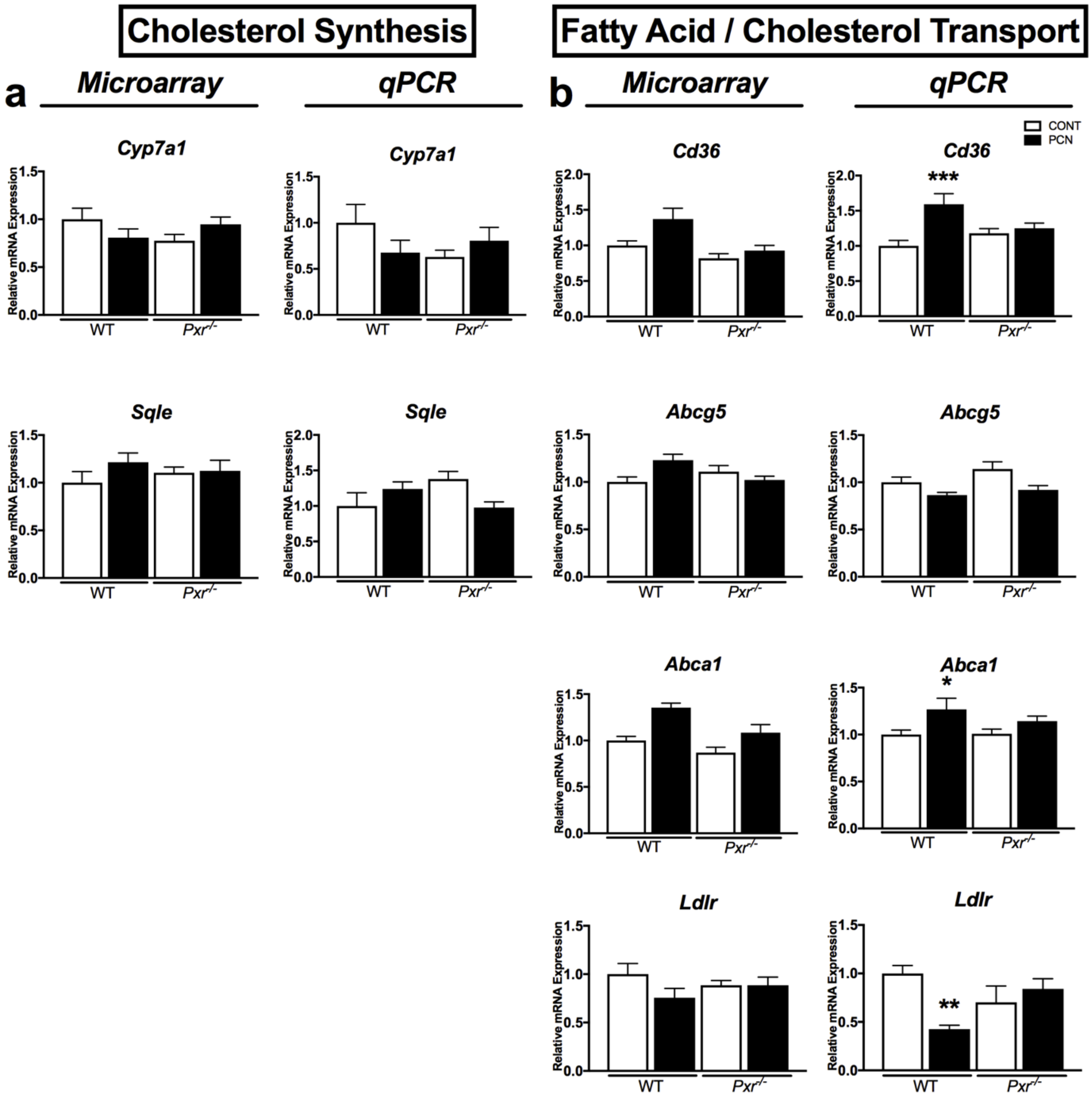

Supplement: Supplementary file 1 [file ijms-20-03767-s001.zip › Supplements/IJMS_Supplements_revised.pdf]
